# A novel reduced-switch asymmetric multilevel inverter with scalable structure and flexible source selection algorithms

**DOI:** 10.1038/s41598-026-44932-4

**Published:** 2026-05-05

**Authors:** Sajad Sabzi, Ebrahim Salary, Mahdi Hedayati, Peyman Hajihosseini

**Affiliations:** Department of Electrical Engineering, Ka.C., Islamic Azad University, Karaj, Iran

**Keywords:** Multilevel inverter, Independent source, Asymmetrical inverter, Reduction of switches, Energy science and technology, Engineering

## Abstract

Multilevel inverter structures, due to their merits such as step voltage generation, reduction of total harmonic distortion, no need for passive filters, reduction of magnetic interference, and ability to withstand high voltages, can be used in various industries such as energy conversion, electric vehicles, and electric machine drives because of these positive features. This work proposes an innovative multi-level inverter architecture designed to overcome constraints inherent in conventional approaches. The structure facilitates the use of either symmetrical or asymmetrical DC sources. Notably, it features a significant reduction in the required number of switches for generating a given number of voltage steps compared to traditional inverters. The possibility of designing an asymmetric inverter with different voltage source selection algorithms is another feature of the proposed structure. For verification, the system performance has been investigated in MATLAB software. Furthermore, an experimental prototype was constructed. Test results confirm the inverter’s capability to produce high-quality voltage waveforms.

## Introduction

The power conversion interface is a critical component in distributed generation (DG) systems, whether supplying loads or connected to the grid. DC-generating sources like fuel cells (FC) and photovoltaics (PV) necessitate an inverter for DC-to-AC power conversion. In the power electronics trend, one of the most influential events is the presence and emergence of inverters, because the presence of these devices was able to significantly affect various sectors of the electricity industry, including renewable energy production, electric drive control, power quality compensation devices, etc.^1–3^. Inverters come in different types depending on their applications. The number of output voltage levels divides inverters into three distinct groups:

1. Two-level inverters
2. Three-level inverters
3. Multi-level inverters

In this paper, multilevel voltage source inverters are discussed. Having evolved for more than 40 years, these converters now represent a critical solution for medium-voltage and high-power implementations across power systems and industry. The output of these inverters consists of multiple voltage levels that are generated by multiple DC voltage sources. DC sources such as batteries, linear power supplies, switching power supplies, floating capacitors, fuel cells, and photovoltaic modules are added together algebraically to generate a stepped output voltage waveform closely approximating an ideal sine wave^4^. Multilevel inverters offer significant advantages over conventional two-level converters:

1. Reducing harmonics.
2. Reducing the switching frequency.
3. Reducing the reverse voltage across the switches.
4. Reducing the dimensions of the filtering system.
5. Reducing the amount of electromagnetic interference.
6. Reducing switching losses.
7. Reducing total harmonic distortion (THD).

Their disadvantages include:

1. Increased control complexity.
2. Increased number of active and passive devices.
3. Unbalance of capacitor voltage during charging and discharging in some structures.
4. Increased possibility of short circuit for DC sources.

Based on transformer implementation, multilevel inverter architectures fall into two groups: transformer-integrated and transformer less configurations^4–6^. The research community has proposed diverse circuit configurations for transformer less multilevel inverters. These types of inverters are usually divided into two general categories: multilevel inverters with a common DC voltage source and with independent sources. The primary multilevel inverter topologies comprise diode-clamped and flying-capacitor configurations (utilizing a centralized DC source), alongside cascaded H-bridge designs (employing isolated DC sources)^7^. In this article, the main discussion focuses on chain structures that use separate DC voltage sources. Multi-level cascade inverters refer to structures that use separate power supplies along with semiconductor elements to create voltage levels. The first member of this group is the cascaded H-bridge inverter. Cascaded H-bridge inverters achieve uniform voltage steps through both symmetric and asymmetric operational modes. Symmetrically configured cascaded inverters consist of uniform H-bridge modules, each utilizing DC sources with identical voltage ratings. Asymmetric implementations, while retaining structural uniformity among the H-bridge modules, incorporate DC voltage sources with non-uniform magnitudes per module. Asymmetric multilevel inverters create a larger number of levels than symmetric structures by using fewer semiconductor switches and DC voltage sources^8^. One of the challenges facing multi-level H-bridge inverters is the high number of semiconductor elements in this type of inverter.

References^6–25^ discuss examples of series inverters that attempt to reduce the number of semiconductors switching elements. In^8–14^, positive or negative voltage polarity is created using separate DC sources and semiconductor switches; then, an H-bridge is placed at the output to create the opposite polarity voltage. In other words, these inverters have two parts: a part that generates voltage with positive or negative polarity and an H-bridge part. This type of structure uses fewer semiconductor switches than H-bridge series inverters. The architectures described in^8–14^ exhibit a critical vulnerability related to switch blocking voltage: switches within each H-bridge module must tolerate the full output voltage potential. References^15,16^ introduce modified configurations specifically designed to mitigate this high-voltage stress on the switches. In^15^ and^16^, the structures proposed in^8^ and^9^ are extended in a chain manner, respectively.

The structure in^17^ introduces a cascaded inverter whose fundamental unit generates nine voltage levels. The topology in^18^ supports both symmetric and asymmetric configurations. Samadaei et al.^19^ employs *n* series-connected sub-inverters, each comprising 4 DC sources, 6 unidirectional, and 3 bidirectional switches. Total output voltage aggregates the levels from all sub-inverters, achieving 17 levels in asymmetric operation. In^20^, a hybrid structure is introduced. The presented structure can operate symmetrically and asymmetrically. Dhanamjayulu et al.^21^ is an example of asymmetric inverters that use a specific algorithm to select sources as 1, 2, and 5. The presented structure is a 15-level structure that uses 8 semiconductor switches. An asymmetric multilevel converter topology is detailed in^22^. The selection of DC sources from numbers 1 to 4 is as follows: 1, 1, 3, and 3, respectively. The presented structure in^22^ can produce 17 voltage levels. Peddapati^23^ suggested a multilevel inverter mighty of operating in both symmetric and asymmetric states. This topology employs nine unidirectional switches to generate voltage levels, although two switches are provided for special fault conditions. In^23^, two methods for selecting DC voltage sources are discussed. This inverter creates 9 levels in the symmetric mode and 17 levels in the asymmetric mode. In^24^, a type of multilevel inverter using a polarity-changing H-bridge structure is introduced. An asymmetric topology in^24^ features an H-bridge for polarity reversal and incorporates separate diodes with semiconductor switches. Critically, some switches in this design omit antiparallel diodes. In^25^, two structures for multilevel inverters are proposed. The inverter topology employs both unidirectional and bidirectional switches. The first structure^25^ in the symmetric case with n separate DC voltage sources is capable of generating 2n-1 levels, which is two levels less than other similar structures. For the asymmetric case, a special method for source selection is proposed. In^26^, an asymmetric 7-level inverter is proposed that uses unidirectional and bidirectional power electronic devices in its structure.

A prominent class of multilevel inverter (MLI) topologies proposed in recent years is the switched-capacitor (SC) structure, with recent examples presented in^27–33^. However, when aiming to maximize the number of output voltage levels while minimizing the component count, cascaded multilevel inverters offer a significant advantage. Specifically, cascaded topologies are capable of generating a higher number of voltage levels compared to SC-MLIs, while utilizing fewer semiconductor switches.

In this paper, structures for multilevel inverters are presented that save costs by reducing the number of devices. The proposed topologies minimize active components within the current conduction path at any instant, reducing switch conduction losses. These inverters inherently support both symmetric and asymmetric operating modes. Optimal selection of DC source magnitudes remains a persistent challenge for asymmetric implementations, and in the proposed structure, various algorithms can be used to select sources. It seems that providing more flexible structures in determining the size of work resources is important. There is a kind of generality and comprehensiveness in the proposed structure for multilevel inverters, so that it can be designed and optimized according to specific goals. For the proposed structure, various indicators, including the number of power electronic devices, DC voltage sources count, the total standing voltage, and the variation in the size of the DC voltage sources, are generally determined using mathematical equations. Following the introduction of the structure, mathematical equations, and optimization of the structure, a design example will be presented for it, and finally, simulation and experimental results will be presented. The proposed inverter is called STUI (Series Two Unit Inverter).

## Proposed multilevel converter

A. Inverter structure

**Fig. 1:**
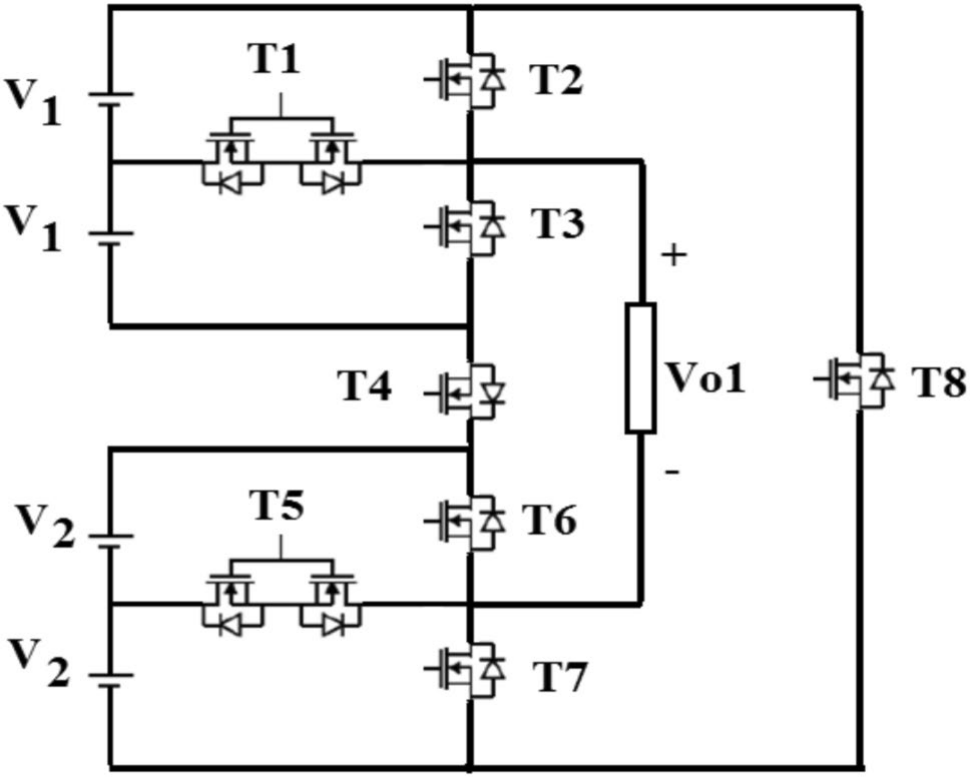
Proposed single phase STUI.

**Fig. 2:**
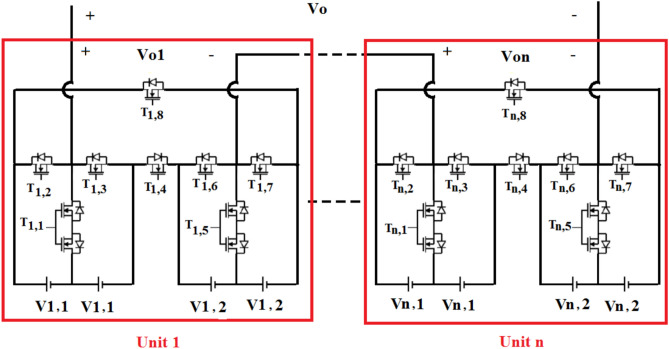
The proposed topology with n inverter units.

Figure 1 illustrates the topology of the STUI circuit, comprising four DC voltage sources along with six unidirectional and two bidirectional semiconductor switches. This diagram represents the fundamental building block for a single-phase implementation. Cascading multiple basic units in series enables the construction of inverters generating increased voltage levels. Furthermore, voltage distribution analysis across semiconductor devices can be extended through this chained configuration. Figure 2 depicts the generalized architecture with *n* series-connected inverter blocks.

The multilevel converter design necessitates both unidirectional and bidirectional power switches, where bidirectional devices are engineered to block voltages and conduct currents in both directions in STUI and consist of two diodes and two power transistors. Semiconductor bidirectional switches prevent short-circuiting of sources.

The phase voltage waveform at the STUI’s output terminals is synthesized by aggregating the output voltages of its constituent basic blocks. Assuming n inverter blocks, the output voltage of Fig. 2 can be written as:

1 $$V_{O} = V_{O1} + V_{O2} + ... + V_{On}$$

The STUI’s operational principle aligns with conventional multilevel H-bridge converters. Similar to series H-bridge topologies, optimal DC source magnitude selection constitutes a critical design consideration for the STUI. Appropriate switching state combinations synthesize positive, negative, and zero voltage states at each basic block. These individual output voltages are summed algebraically across the series chain. Fundamentally, the aggregate output voltage results from the coordinated operation of single-phase basic inverters, equalling the summation of all sub-inverter contributions. Table 1 illustrates the performance of the STUI of Fig. 1 for generating different voltage levels. The peak of the output voltage is determined by the size of the input DC voltage sources. Operating in either symmetric or asymmetric configurations, the STUI achieves uniform voltage-level stepping across its output waveform.

**Table 1:** Lookup table switching.

On switches	Vo	On switches	Vo
T_3_, T_4_, T_6_	0	T_2_, T_7_, T_8_	0
T_1_, T_4_, T_6_	V_1_	T_1_, T_7_, T_8_	− V_1_
T_2_, T_4_, T_6_	2V_1_	T_3_, T_7_, T_8_	− 2V_1_
T_3_, T_4_, T_5_	V_2_	T_2_, T_5_, T_8_	− V_2_
T_3_, T_4_, T_7_	2 V_2_	T_2_, T_6_, T_8_	− 2V_2_
T_1_, T_4_, T_5_	(V_1_ + V_2_)	T_1_, T_5_, T_8_	− (V_1_ + V_2_)
T_2_, T_4_, T_5_	(2V_1_ + V_2_)	T_3_, T_5_, T_8_	− (2V_1_ + V_2_)
T_1_, T_4_, T_7_	(V_1_ + 2V_2_)	T_1_, T_6_, T_8_	− (V_1_ + 2V_2_)
T_2_, T_4_, T_7_	(2V_1_ + 2V_2_)	T_3_, T_6_, T_8_	− (2V_1_ + 2V_2_)

As shown in Table 1, three semiconductor switches are on to create each level at any given moment, which can be effective in reducing losses. In a case comparison, a 9-level H-bridge symmetric series inverter requires a total of six switches to create each level.

In the STUI structure, the count of semiconductor switches in terms of the number of inverter units is equal to:

2 $$N_{sw} = 8n$$

B. Symmetric and asymmetric STUI

A multilevel inverter operates symmetrically when all DC voltage sources exhibit identical magnitudes. The maximum number of phase voltage levels is:

3 $$m = 8n + 1$$

The parameters *n* and *m* denote the quantity of base blocks and the maximum phase voltage levels, respectively. For instance, with four DC sources per block and two series-connected blocks (*n* = 2), the topology yields 17 voltage levels. When all sources share identical voltage magnitude *V*_*dc*_, the peak output voltage, *V*_*O*max_ is given by:

4 $$V_{O\max } = 4nV_{dc}$$

In the STUI structure, the number of semiconductor switches in terms of the number of levels is equal to:

5 $$N_{sw} = m - 1$$

Asymmetric multilevel converters enable enhanced output levels without augmenting component count. In the STUI topology, asymmetric operation employs varied DC source magnitudes and configurable strategies. This work proposes nine distinct methodologies to optimize DC voltage source magnitudes across *n* series-connected basic blocks. The existence of different algorithms for determining the transformer turns ratio gives the designer more freedom to design the multilevel inverter. In the whole method, assume that:

6 $$V_{1,1} = V_{dc}$$

Nine distinct methodologies for DC voltage source selection are analyzed. Subsequent sections derive the following relationships:

(i) Voltage levels *m* as a function of base block count *n*,
(ii) Switch count *Nsw* versus output levels *m*,
(iii) Peak phase voltage, *V*_*o*max_ relative to inverter units *n*.

Method 1: In the first algorithm, to obtain a uniform step, it is proposed to select the voltage magnitude of the DC sources according to a geometric progression with a magnitude of 2. In this algorithm, the voltage magnitude of the DC sources is selected according to the following equations.

7 $$V_{i,1} = V_{i,2} = 2^{i - 1} V_{dc} ,\;i = 1,2,...,n$$

8 $$m = 2^{n + 3} - 7$$

9 $$N_{sw} = 8\left( {\frac{Ln(m + 7)}{{Ln2}} - 3} \right)$$

10 $$V_{O\max } = (2^{n + 2} - 4)V_{dc}$$

Method 2: Geometric progression with a magnitude of 3

11 $$V_{i,1} = V_{i,2} = 3^{i - 1} V_{dc} ,\;i = 1,2,...,n$$

12 $$m = 4 \times 3^{n} - 3$$

13 $$N_{sw} = 8\left( {\frac{Ln(m + 3) - Ln4}{{Ln3}}} \right)$$

14 $$V_{O\max } = 2(3^{n} - 1)V_{dc}$$

Method 3: Geometric progression with a magnitude of 4

15 $$V_{i,1} = V_{i,2} = 4^{i - 1} V_{dc} ,\;i = 1,2,...,n$$

16 $$m = \frac{8}{3}(4^{n} - 1) + 1$$

17 $$N_{sw} = 8\left( {\frac{Ln(3m + 5) - Ln8}{{Ln4}}} \right)$$

18 $$V_{O\max } = \frac{4}{3}(4^{n} - 1)V_{dc}$$

Method 4: Geometric progression with a magnitude of 5

19 $$V_{i,1} = V_{i,2} = 5^{i - 1} V_{dc} ,\;i = 1,2,...,n$$

20 $$m = 2 \times 5^{n} - 1$$

21 $$N_{sw} = 8\left( {\frac{Ln(m + 1) - Ln2}{{Ln5}}} \right)$$

22 $$V_{O\max } = (5^{n} - 1)V_{dc}$$

Method 5: Geometric progression with a magnitude of 6

23 $$V_{i,1} = V_{i,2} = 6^{i - 1} V_{dc} ,\;i = 1,2,...,n$$

24 $$m = \frac{8}{5}(6^{n} - 1) + 1$$

25 $$N_{sw} = 8\left( {\frac{Ln(5m + 3) - Ln8}{{Ln6}}} \right)$$

26 $$V_{O\max } = \frac{8}{10}(6^{n} - 1)V_{dc}$$

Method 6: Geometric progression with a magnitude of 7

27 $$V_{i,1} = V_{i,2} = 7^{i - 1} V_{dc} ,\;i = 1,2,...,n$$

28 $$m = \frac{4}{3}(7^{n} - 1) + 1$$

29 $$N_{sw} = 8\left( {\frac{Ln(3m + 1) - Ln4}{{Ln7}}} \right)$$

30 $$V_{O\max } = \frac{2}{3}(7^{n} - 1)V_{dc}$$

Method 7: Geometric progression with a magnitude of 8

31 $$V_{i,1} = V_{i,2} = 8^{i - 1} V_{dc} ,\;i = 1,2,...,n$$

32 $$m = \frac{8}{7}(8^{n} - 1) + 1$$

33 $$N_{sw} = 8\left( {\frac{Ln(7m + 1) - Ln8}{{Ln8}}} \right)$$

34 $$V_{O\max } = \frac{4}{7}(8^{n} - 1)V_{dc}$$

Method 8: Geometric progression with a magnitude of 9

35 $$V_{i,1} = V_{i,2} = 9^{i - 1} V_{dc} ,\;i = 1,2,...,n$$

36 $$m = 9^{n}$$

37 $$N_{sw} = 8\left( {\frac{Lnm}{{Ln9}}} \right)$$

38 $$V_{O\max } = \frac{1}{2}(9^{n} - 1)V_{dc}$$

Method 9: The Ninth algorithm employs a heuristic approach where DC voltage sources within each inverter unit exhibit non-uniform magnitudes. This method maximizes achievable voltage levels under asymmetric operation.

39 $$V_{i,1} = 17^{i - 1} V_{dc} ,\;i = 1,2,...,n$$

40 $$V_{i,2} = 3 \times 17^{i - 1} V_{dc} ,\;i = 1,2,...,n$$

41 $$m = 17^{n}$$

42 $$N_{sw} = 8\left( {\frac{Lnm}{{Ln17}}} \right)$$

43 $$V_{O\max } = \frac{1}{2}(17^{n} - 1)V_{dc}$$

As mentioned, there are various algorithms for the asymmetric STUI structure that allow for the creation of a stepped voltage with a uniform step. In addition to algorithms with specific relationships, the STUI structure can also use arbitrary methods in selecting voltage sources.

C. Modulation strategy

**Fig. 3:**
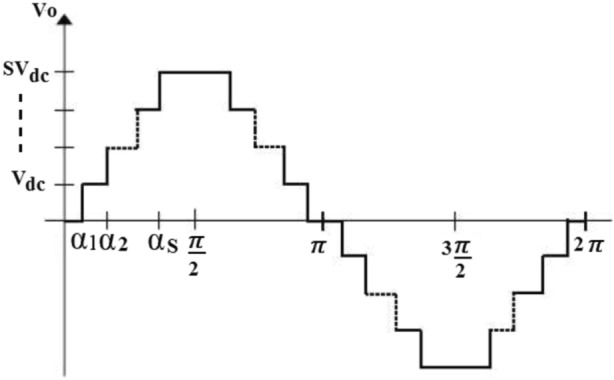
m-step voltage.

**Fig. 4:**
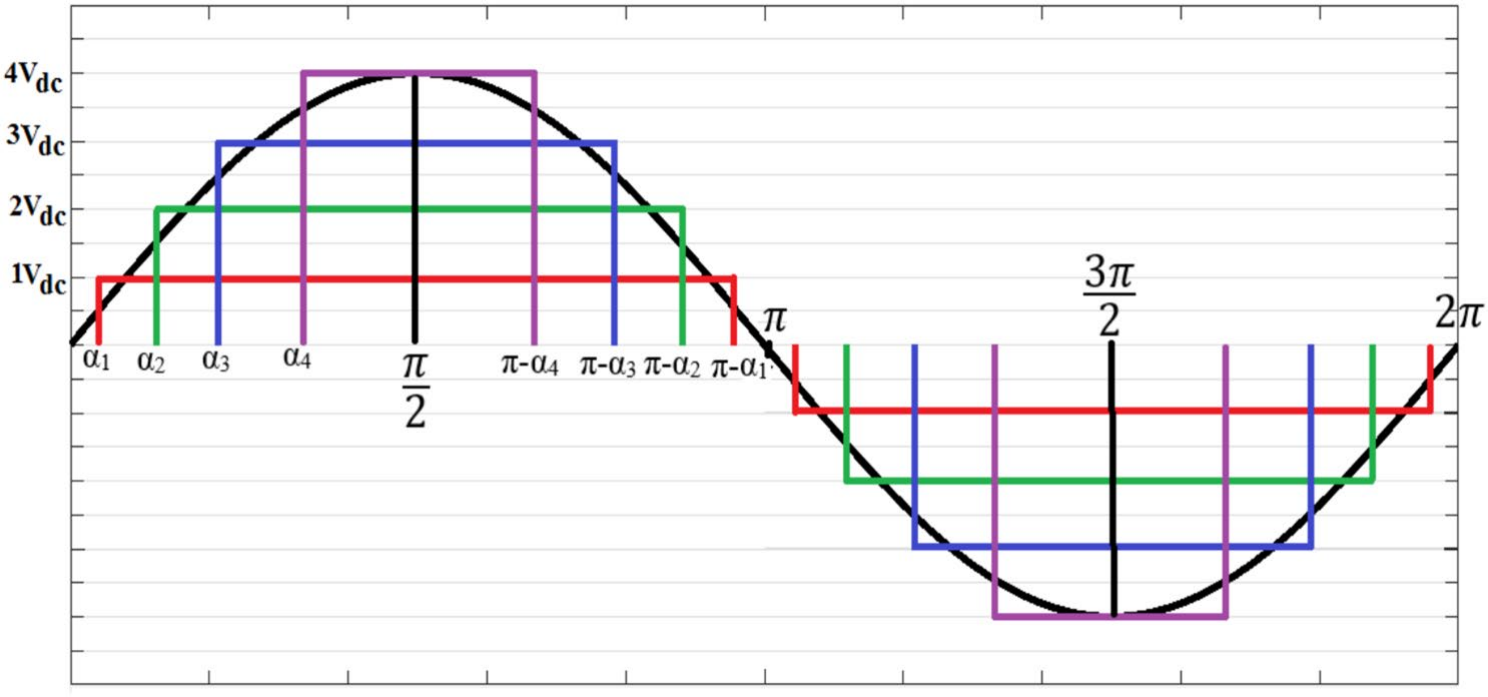
How to create a step voltage with a uniform step and switching angles.

Multilevel converter switching strategies are broadly categorized as high-frequency or fundamental-frequency methods^34,35^. This work adopts the fundamental frequency technique, offering reduced switching losses and operational advantages^26^. For an m-level output voltage (Fig. 3) with maximum magnitude *SV*_*dc*_, voltage synthesis requires determination of switching angles (α_1_, α_2_,…, α_S_) through established switching tables. Figure 4 shows how to create a step voltage with a uniform step and switching angles. The difference in switching angles gives the pulses necessary to create the voltage levels.

Assuming a step voltage with uniform step for the fundamental and non-fundamental harmonics, Eq. (44) is given.

44 $$H_{i} = \left\{ {\begin{array}{*{20}l} {\frac{{4V_{{dc}} }}{{i\pi }}\sum\nolimits_{{j = 1}}^{S} {\cos (i\alpha _{j} )} } \hfill & {{\mathrm{For}}\;{\mathrm{odd}}\;{\mathrm{n}}} \hfill \\ 0 \hfill & {{\mathrm{For}}\;{\mathrm{even}}\;{\mathrm{n}}} \hfill \\ \end{array} } \right.$$

In (44), *H*_*i*_ represents the magnitude of the *i*-*th* harmonic component of the output voltage. The term *V*_*dc*_ denotes the voltage of one step in the multilevel output waveform. The index *j* corresponds to the *j-th* switching angle, with *α*_*j*_ representing the specific angle. The index *i* indicates the harmonic order, and the conditional statement (odd/even *n*) refers to the same harmonic order *i*, indicating that even-order harmonics are zero due to half-wave symmetry. The summation index *j* runs from 1 to *S. S* is the highest voltage level created, for example for a nine-level inverter *S* is equal to 4. In general, for an m-level inverter the value of *S* is equal to:

45 $$S = \frac{m - 1}{2}$$

Equation (46) can be used to determine the switching angles.

46 $$\alpha_{j} = \sin^{ - 1} \left( {\frac{j - 0.5}{S}} \right),\;j = 1,2,...,S$$

where $$\alpha_{1} < \alpha_{2} < ... < \alpha_{S} < \frac{\pi }{2}$$.

The output voltage total harmonic distortion (THD) is determined by:

47 $$THD = \frac{{\sqrt {\sum\nolimits_{h = 3,5,..}^{\infty } {V_{h}^{2} } } }}{{V_{1} }}$$

where *V*_*h*_ is the *h-th* order harmonic and V_1_ is a fundamental voltage harmonic.

D. Voltage stress

Semiconductor pricing correlates with switch voltage/current specifications. For cascaded inverters, switch current is load-dependent, with maximum current stress equalling the load current. Consequently, switches require a minimum current rating matching the peak load current. But regarding the maximum switch voltage, the maximum voltage applied across the two ends of the semiconductor switch when the switch is off depends on the inverter structure. Semiconductor switches with higher voltage tolerances are more expensive than other switches. The voltage stress experienced by switches is governed by DC source voltage magnitudes in each inverter module. Mathematically, the switch voltage stress *VS*​ is expressed by:

48 $$VS_{Tn,1} = V_{n,1}$$

49 $$VS_{Tn,2} = VS_{Tn,3} = 2V_{n,1}$$

50 $$VS_{Tn,4} = 2V_{n,1} + 2V_{n,2}$$

51 $$VS_{Tn,5} = V_{n,2}$$

52 $$VS_{Tn,6} = VS_{Tn,7} = 2V_{n,2}$$

53 $$VS_{Tn,8} = 2V_{n,1} + 2V_{n,2}$$

Suppose that the peak inverse voltage (PIV) of all switches is defined as follows to compare the voltage stress of converters^8^.

54 $$PIV = \sum\limits_{i = 1}^{{N_{SW} }} {VS_{i} }$$

here N_SW_ and VS are the number of switches and the maximum voltage blocked by the switches, respectively.

E. Inverter loss

Elements such as inductors, capacitors, and semiconductors have internal resistance. Semiconductors also have a voltage drop in the conduction state. These types of resistances and voltage drops create ohmic losses or conduction losses for the converter. Another type of converter loss is related to the switching operation, which is known as switching losses^22–25^. Per switching cycle, the conduction losses associated with the semiconductor switches (*P*_*C*,*T*_) and the antiparallel diodes (*P*_*C*,*D*_) are calculated using:

55 $$P_{C} = P_{C,T} + P_{C,D}$$

56 $$P_{C} = \int_{0}^{2\pi } {[Z_{1} (t)[(V_{D} + R_{D} I_{P} (t))]} + Z_{2} (t)[(V_{T} + R_{T} I_{P}^{\beta } )]I_{P} ]d(\omega t)$$

where V_T_: Forward voltage drop across the transistor during conduction, V_D_: Forward voltage drop across the antiparallel diode during conduction, R_T_: On-state resistance of the transistor, R_D_: On-state resistance of the diode, β: Device-specific constant for the transistor, I_P_: Peak magnitude of the output current waveform.

The values of *Z*_1_(*t*) and *Z*_2_(*t*) can be different according to each output voltage levels of the proposed multilevel inverter.

The converter’s switching losses are determined by the switching frequency, switch transition times (turn-on/off), and the number of conducting switches required to synthesize a specific voltage level. The energy dissipated during switch commutation (turn-on and turn-off) is expressed as follows^25^:

57 $$E_{on,j} = \int_{0}^{{t_{on} }} {v(t)i(t)} dt = \frac{1}{6}V_{SW,j} \times I \times t_{on}$$

where E_on,j_: Energy dissipated during the turn-on commutation of switch *j*, t_on_ : turn-on time of the switch. I: Instantaneous current flowing through the switch once conduction is established.

58 $$E_{off,j} = \frac{1}{6}V_{SW,j} \times I{\prime} \times t_{off}$$

where E_off,j_: Energy dissipated during turn-off commutation of switch *j*,

t_off_: The turnoff time of the switch, $I{\prime}$: Current flowing through switch *j* immediately prior to turn-off initiation, V_sw,j_: Voltage blocked by switch *j* during its non-conducting state.

Commutation losses accumulate proportionally to switching frequency. The mean switching power loss is generally given by^25^:

59 $$P_{SW} = f_{S} \left[ {\sum\limits_{j = 1}^{{N_{SW} }} {\left[ {\sum\limits_{i = 1}^{{N_{on,j} }} {E_{on,ji} } + \sum\limits_{i = 1}^{{N_{off,j} }} {E_{off,ji} } } \right]} } \right]$$

where *N*_*on*,*j*_: Count of turn-on commutations for switch *j* over one half-cycle of the fundamental output period, *N*_*off,j*_: Count of turn-off commutations for switch *j* over the same half-cycle interval, *f*_*s*_: Operating frequency at which the power semiconductor devices commutate, *E*_*on*,*ji*_: Energy dissipated during the *i*-th turn-on event of switch *j*, *E*_*off*,*ji*_: Energy dissipated during the *i*-th turn-off event of switch *j*.

Switching losses are directly related to switching frequency, so reducing the switching frequency using fundamental frequency modulation can be effective in reducing switching losses. Then total losses of inverter are given as:

60 $$P_{Loss} = P_{c} + P_{sw}$$

Loss reduction in multilevel inverters is achievable by minimizing the total semiconductor devices in the topology, and the number of conducting switches required to synthesize each voltage level. What is certain is that the efficiency of converters in practice also depends on the type and material of the components and not only on the shape of the structure. Also, losses depend on temperature conditions and the use of peripheral circuits.

The proposed 17-level asymmetric inverter consists of 8 power switches and can produce 17 voltage levels. This topology is thermally modeled in PLECS software to assess power losses and efficiency. The IRFP460 MOSFET is utilized in this investigation, corresponding to the experimental prototype. The resistance of drain source is 0.27Ω. The inverter’s input is connected via four regulated DC sources with voltages selected according to the asymmetric algorithm. The inverter output is connected to a pure resistance of 80 Ω to provide 1 kW of power with a peak output voltage of 400 V.

The power loss of each switch is analyzed, considering both conduction and switching losses. Due to the use of fundamental frequency modulation, the switching losses are very low. At any given instant, only three switches conduct simultaneously to synthesize the output voltage level, which minimizes conduction losses. Among these, one switch is bidirectional, which exhibits slightly higher power loss than unidirectional switches because it consists of two anti-series MOSFETs, and both devices contribute to the conduction path. The total power loss of the proposed 17-level inverter at 1 kW output power is calculated as 21W. Consequently, the efficiency of the inverter at 1 kW output power is 97.8%, confirming that the proposed STUI topology achieves high efficiency while maintaining low component count (only 8 switches) and high output quality (17-level waveform).

F. Comparison study

This section provides a comprehensive comparison between the proposed multilevel inverter structure, recent state-of-the-art topologies (published within the last five years). Key performance metrics include:

- Total active switch count
- Semiconductor device requirement
- Maximum achievable output voltage levels
- Number of conducting switches per output voltage level

Given that the main goal of the article is to present new multi-level cascade inverters with a component reduction approach, the main discussion of the comparison focuses on the number of switches. One of the most important components that has the greatest impact on the cost of the converter is the number of controllable semiconductor switches. Since each active switch necessitates a dedicated gate driver circuit. The price of the driver circuit may be higher than that of the semiconductor switch. The total driver count directly lowers system expenses. Minimizing drivers thus becomes critical for cost-optimized multilevel converter designs. On the other hand, switches also require protection and cooling circuits. Furthermore, higher switch counts exponentially increase control system complexity. Therefore, in general, it can be said that with the increase in the number of controllable semiconductors and their driver circuits, the price of the converter increases. To systematically evaluate the performance trade-offs and advantages of the STUI inverter, Table 2 and 3 present a comparison study between the proposal and other recent multilevel inverters^19–26^. Due to the inherent differences between symmetric and asymmetric configurations, the comparison is presented in two separate tables. Table 2 compares symmetric topologies, while Table 3 compares asymmetric topologies. For the proposed structure, the values for the asymmetric inverter are provided using the ninth DC voltage source selection algorithm. Some references have presented an inverter unit for multi-level inverters with the approach of reducing the number of switches, and there has been no discussion of expanding the structure in a chain manner. The ratio of the number of levels per switch and the ratio of the number of levels per number of semiconductor switches are defined as LSR and LSEMR, respectively^26^. In comparing two inverters, the larger the LSR or LSEMR ratios, the more the inverter has produced higher voltage levels with fewer switches or semiconductor switches. According to Table 2 and 3, the presented structure is in good condition in terms of LSR and LSEMR indices. For example, the LSR index for 17-level inverters^19,22^, and^23^ is calculated to be 1.888, 1.7, and 1.416, respectively, while for the proposed 17-level structure it is 2.125. According to Tables 2 and 3, compared to^25^, in the case where the number of switches and semiconductor switches for both structures is 16 and 20, the STUI creates a higher number of levels.

**Table 2:** Comparison study in symmetric topology.

	N_L_	N_SEM_	N_SW_	LSEMR	LSR	N_DC_	N_ONSW_	PIV
^19^	9	12	9	0.75	1	4	3	40 E
^23^	9	12	12	0.75	0.75	4	4	36 E
^25^First proposed	11	12	10	0.916	1.1	6	3	38.4 E
^25^Second proposed	11	18	16	0.611	1.833	6	3–7	38.4 E
STUI	9	10	8	0.9	1.125	4	3	36 E

**Table 3:** Comparison study in asymmetric topology.

	N_L_	N_SEM_	N_SW_	LSEMR	LSR	N_DC_	N_ONSW_	PIV
^19^	17	12	9	1.416	1.888	4	3	40 E
	289	24	18	12.041	16.055	8	6	40 E
^20^	15	10	9	1.5	1.666	3	4	35.428 E
^21^	15	8	8	1.875	1.875	3	4	32 E
^22^	17	9	10	1.888	1.7	4	4	33 E
^23^	17	12	12	1.416	1.416	4	5	36 E
^24^	13	10	10	1.3	1.3	3	3–5	45.333 E
	25	16	16	1.562	1.562	6	4–8	45.333 E
^25^First proposed	35	12	10	2.5	3.5	6	3	38.4 E
	51	14	16	3.64	3.187	8	3	38.4 E
^25^Second proposed	63	18	16	3.5	3.937	6	3–7	38.4 E
	255	20	16	12.75	15.937	8	3–9	38.4 E
^26^	7	8	6	0.875	1.166	2	2	42.666 E
STUI	17	10	8	1.7	2.125	4	3	36 E
	289	20	16	14.45	18.0625	8	6	36 E

In multilevel inverters, synthesizing each output voltage level requires configuring a specific combination of semiconductor switches into their conduction states, thereby establishing controlled current paths through the topology. The fewer these switches are, the better the inverter will be in terms of conduction state losses. In the table, N_ONSW_ refers to the number of switches turned on to create a voltage step. Column N_ONSW_ in Tables 2 and 3 shows the number of on-state switches for the inverters being compared, given that in some inverters, different voltage levels are created with different numbers of switches. According toTables 2 and 3, the structures^19,25^, and the proposed one have a more favourable situation in terms of the number of on-state switches than the other structures.

Another parameter used to compare presented topologies is the PIV of the switches. In some references, TSV(Total Standing Voltage) is used for the total maximum voltage applied to the inverter switches. Column PIV inTables 2 and 3 is dedicated to the total maximum reverse voltage of the switches for the inverters under study. InTables 2 and 3, for a more accurate comparison, it is assumed that all inverters have a maximum voltage of 8E. According to Table 2, in terms of the PIV index, structures^21^ and^22^ are in a better position than other structures. It should be noted that the difference in the PIV index between the proposed structure and^21^ or^22^ is not very large. Also, the STUI uses a smaller number of switches than^21^ and^22^.

A prominent class of multilevel inverter (MLI) topologies proposed in recent years is the switched-capacitor (SC) type^27–32^. The primary advantage of SC structures over the proposed topology is their reduced reliance on independent DC voltage sources, as they utilize capacitors to achieve higher voltage levels. As a primary objective of this work is to minimize the number of semiconductor components, a general comparison between the proposed structure and several recent SC MLIs is presented in Table 4. For this comparison, each bidirectional switch is considered equivalent to two semiconductors. The parameters N_Cap_ and N_D_ denote the number of capacitors and independent diodes, respectively. Table 4 demonstrates that the proposed structure generates a higher number of voltage levels compared to the SC topologies, while utilizing fewer switches and semiconductors. Furthermore, the proposed STUI structure inherently eliminates the need for both independent diodes and external capacitors.

**Table 4:** Comparison between the STUI and switched capacitor structures.

	N_L_	N_SEM_	N_SW_	N_D_	N_Cap_	N_DC_
^27^	9	12	10	1	2	1
^28^	9	12	10	–	2	2
^29^	9	10	8	4	2	2
^30^	7	10	9	1	4	1
^31^	13	12	12	2	3	1
^32^	5	6	6	2	1	1
STUI	9	10	8	–	–	4
	17	10	8	–	–	4

## Simulation and experimental results

**Fig. 5:**
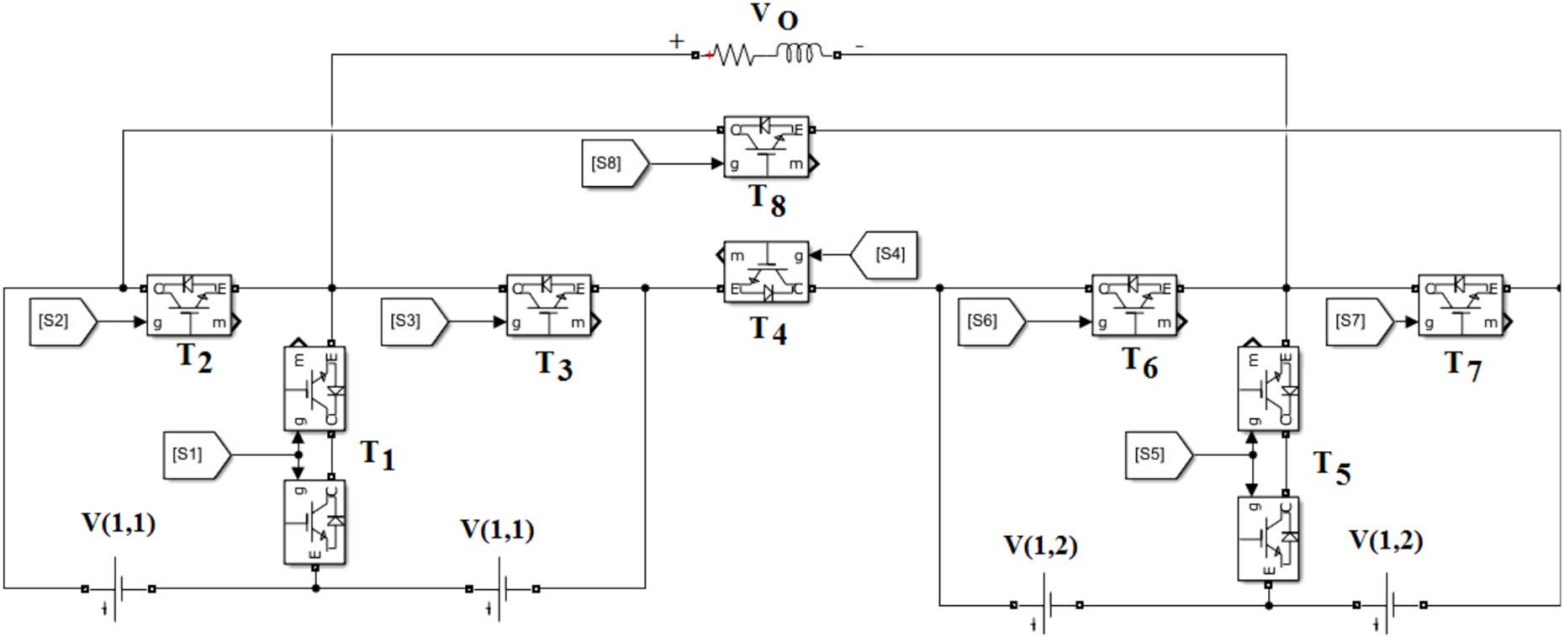
Circuit of STUI in MATLAB/SIMULINK software.

**Fig. 6:**
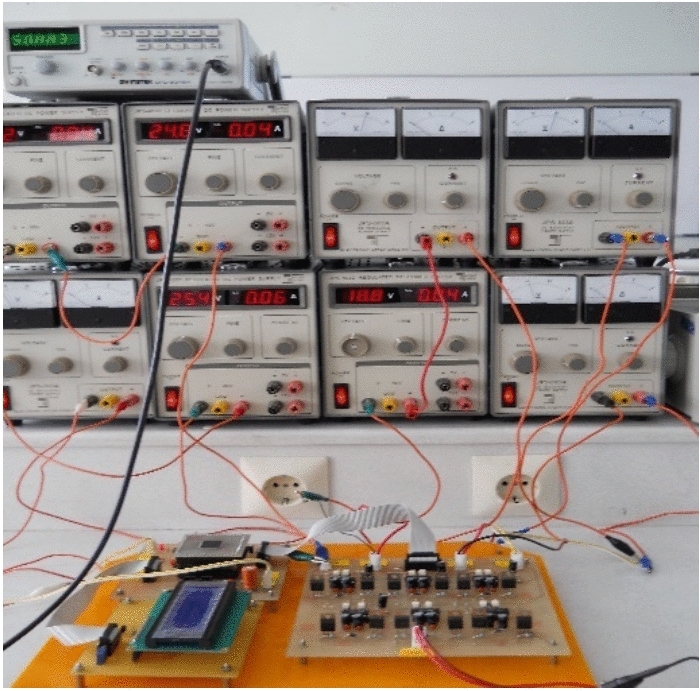
Experimental setup.

To validate the voltage waveform quality and performance characteristics of the proposed symmetric/asymmetric multilevel inverter, MATLAB/Simulink simulations were executed under standardized test conditions (Fig. 5). Experimental verification was subsequently performed using the laboratory prototype shown in Fig. 6, with key components detailed in Table 5. The gating signals for the power switches were generated using an Atmega64 microcontroller from the AVR family. This microcontroller was programmed to produce PWM pulses based on the pre-calculated switching angles obtained from the fundamental frequency switching technique described in Sect. “Proposed multilevel converter”. C. A dead-time of 2 µs was implemented in software to prevent shoot-through faults. The switching angles were stored in look-up tables within the microcontroller’s flash memory, and the pulse outputs were generated using the microcontroller’s built-in timer/counter modules. The gate driver ICs (TLP250) were used to amplify the microcontroller’s output signals and provide electrical isolation between the control and power stages. The experimental prototype utilized IRFP460 MOSFETs as the main switching devices, and the load consisted of a 50 Ω resistor in series with a 100 mH inductor.

**Table 5:** Experimental parameters.

Parameter	Value
Output frequency	50 Hz
Switch	IRFP460
Gate driver	TLP250
Microcontroller	Atmega64
Load	R = 50 Ω and L = 100 mH

A. Symmetric state

**Fig. 7:**
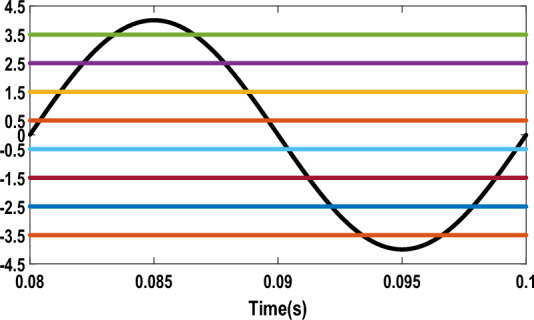
The modulation for calculating the switching angles of 9-level inverter.

This section validates the proposed 9-level inverter’s capability to generate a stepped sinusoidal output with:

- Peak voltage: 196 V
- Fundamental frequency: 50 Hz
- Uniform voltage step: 48 V per level

through MATLAB/Simulink simulation and laboratory prototyping. Figure 7 details the fundamental switching frequency modulation scheme used to calculate switching angles, given by:

$$\alpha_{1} = 7.18.08$$, $$\alpha_{2} = 22.0243$$, $$\alpha_{3} = 36.6822$$ and $$\alpha_{4} = 61.045$$.

**Fig. 8:**
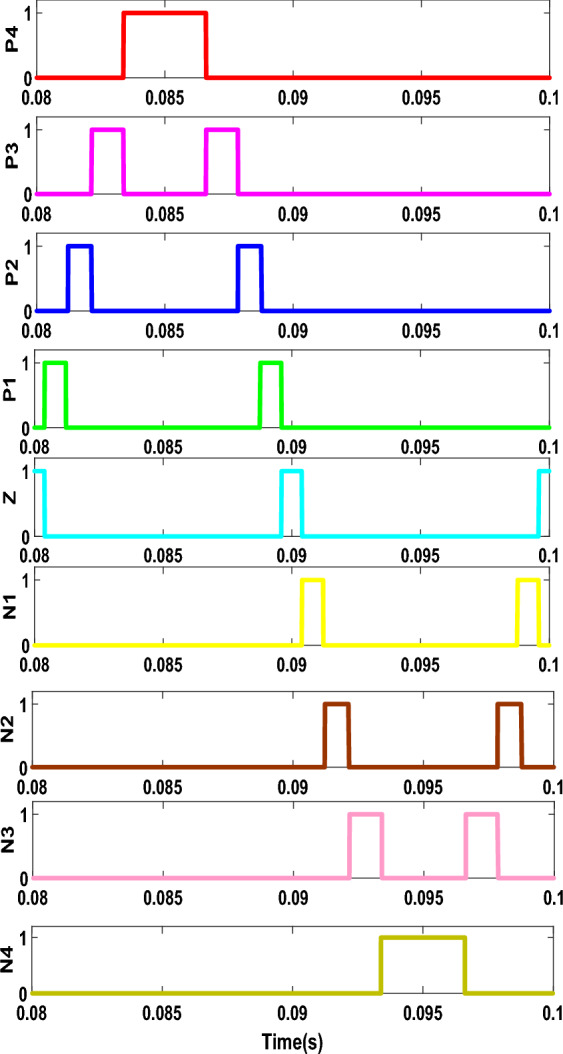
The switching pulses for creating a step voltage.

Figure 8 shows the switching pulses for creating a step voltage. Switching pulses are created from the difference in switching angles with each other or by 90º. For example, pulse P2 is created from the difference in angles $\alpha_{2}$ and $\alpha_{3}$. If the voltage of all DC sources is considered equal to 48, the lookup table for the STUI is as shown in Table 6. In this case, the inverter is symmetric with 9 levels.

**Table 6:** Lookup table of 9-level inverter.

On switches	Vo(V)	On switches	Vo(V)
T_3_, T_4_, T_6_	0	T_2_, T_7_, T_8_	0
T_1_, T_4_, T_6_	48	T_1_, T_7_, T_8_	− 48
T_2_, T_4_, T_6_	96	T_3_, T_7_, T_8_	− 96
T_2_, T_4_, T_5_	144	T_3_, T_5_, T_8_	− 144
T_2_, T_4_, T_7_	192	T_3_, T_6_, T_8_	− 192

**Fig. 9:**
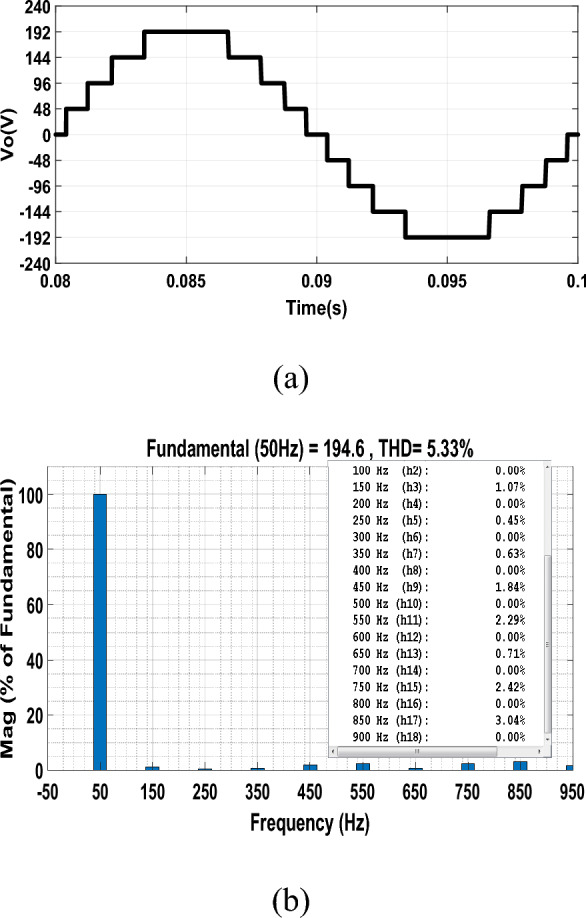
Output voltage. **a** Waveform, **b** FFT analysis.

**Fig. 10:**
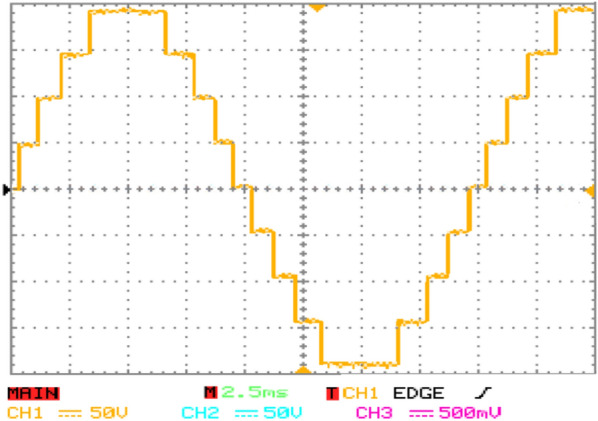
Measured output voltage (50V/Div and 2.5 ms/Div).

Figure 9 demonstrates the output voltage of the 9-level STUI along with its Fourier analysis. The creation of a 9-level voltage with a uniform step of 48 V is clearly evident in Fig. 9. Using Fourier analysis, the total harmonic distortion was calculated to be 5%. The output voltage of the laboratory sample is represented in Fig. 10. Figure 8 confirms the laboratory prototype’s capability to generate the target 9-level staircase waveform at 50 Hz with peak voltage 196 V.

**Fig. 11:**
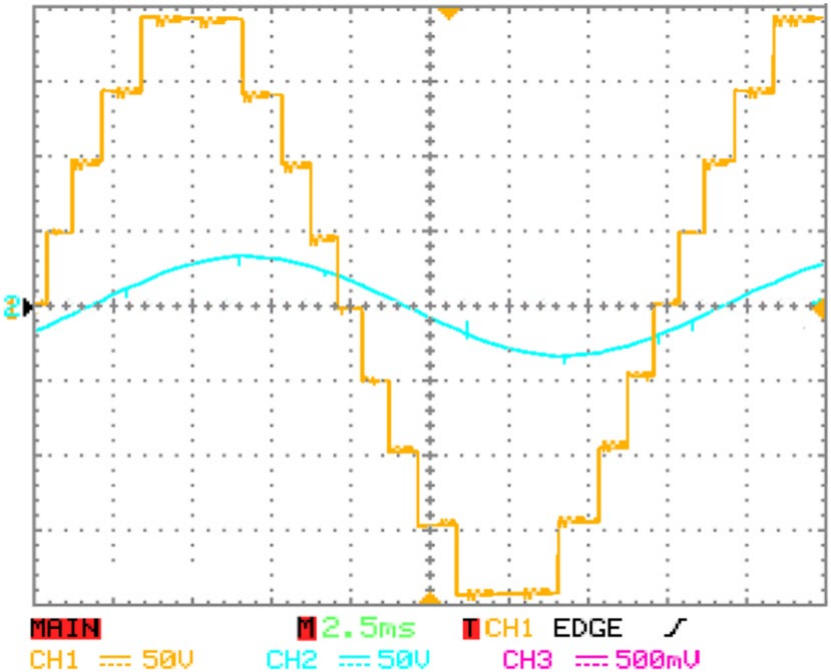
Both the load voltage and current (50V/Div, 50A/Div and 2.5 ms/Div).

The load voltage and current are plotted side-by-side in Fig. 11. To make the current waveform easier to distinguish, it has been amplified by a factor of 10. The phase difference, where the current lags the voltage, results from the load’s ohmic-inductive characteristics.

B. Asymmetric state

As mentioned, there are different algorithms for selecting DC voltage sources in STUI inverters. In this part, the ninth algorithm for selecting DC voltage sources in the asymmetric mode has been selected to study the asymmetric mode. The voltage sources are chosen as follows:

$$V_{1,1} = 24V$$ and $$V_{1,2} = 72V$$.

**Fig. 12:**
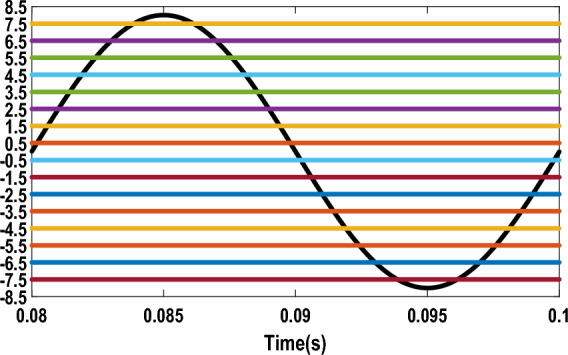
The modulation for calculating the switching angles of 17-level inverter.

Figure 12 shows the modulation for calculating the switching angles. The switching angles are:

$$\alpha_{1} = 3.583$$, $$\alpha_{2} = 10.806$$, $$\alpha_{3} = 18.21$$, $$\alpha_{4} = 25.944$$, $$\alpha_{5} = 34.228$$, $$\alpha_{6} = 43.432$$, $$\alpha_{7} = 54.34$$ and $$\alpha_{8} = 69.635$$.

**Fig. 13:**
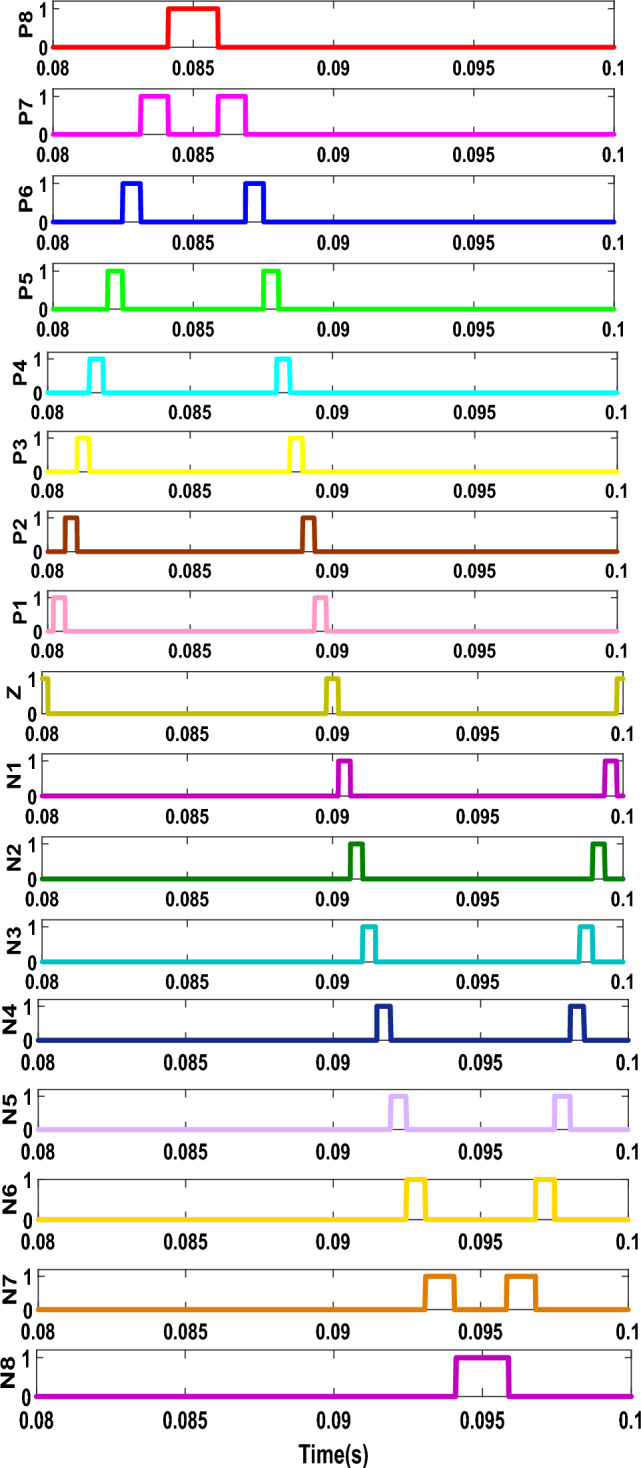
The switching pulses for creating a step voltage.

**Fig. 14:**
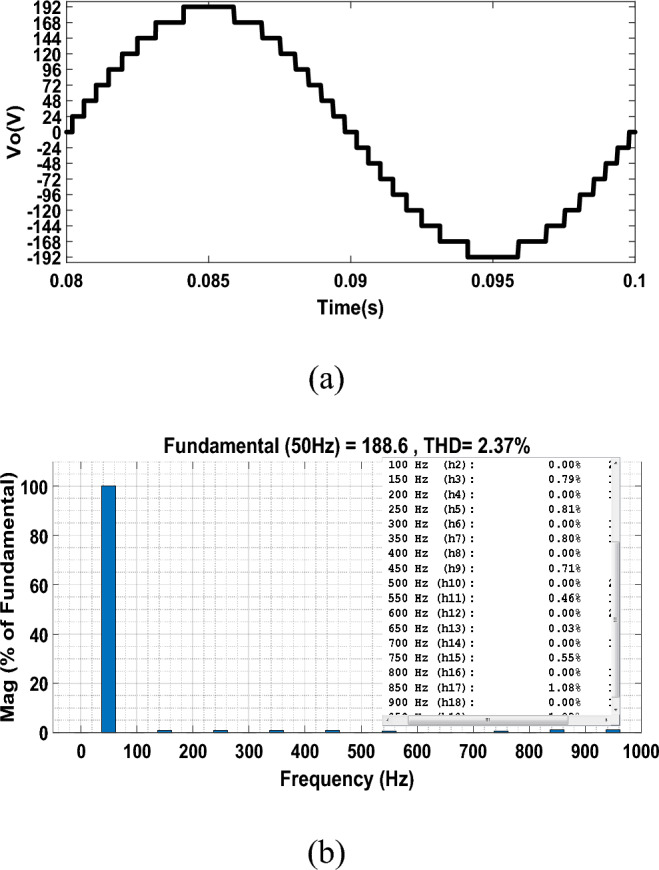
Output voltage. (**a**) Waveform, (**b**) FFT analysis.

**Fig. 15:**
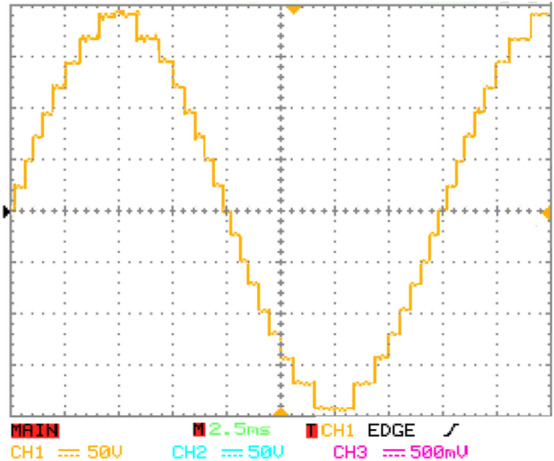
Measured output voltage 17-level (50V/Div and 2.5ms/Div).

Figure 13 shows the switching pulses to create a 17-level voltage. Table 7 describes the switching table of the 17-level STUI inverter. Figure 14 depicts the output voltage and it’s FFT analysis. This is a 50 Hz staircase waveform with amplitude 192 V. The creation of a 17-level voltage with a uniform step of 24 V is clearly evident in Fig. 14. According to Fourier analysis, the THD value is 2%. Figure 15 displays the measured output voltage, confirming the prototype inverter’s capability to generate the target waveforms.

**Table 7:** Lookup table of 17-level inverter

On Switches	Vo(V)	On Switches	Vo(V)
T_3_, T_4_, T_6_	0	T_2_, T_7_, T_8_	0
T_1_, T_4_, T_6_	24	T_1_, T_7_, T_8_	− 24
T_2_, T_4_, T_6_	48	T_3_, T_7_, T_8_	− 48
T_3_, T_4_, T_5_	72	T_2_, T_5_, T_8_	− 72
T_1_, T_4_, T_5_	96	T_1_, T_5_, T_8_	− 96
T_2_, T_4_, T_5_	120	T_3_, T_5_, T_8_	− 120
T_3_, T_4_, T_7_	144	T_2_, T_6_, T_8_	− 144
T_1_, T_4_, T_7_	168	T_1_, T_6_, T_8_	− 168
T_2_, T_4_, T_7_	192	T_3_, T_6_, T_8_	− 192

**Fig. 16:**
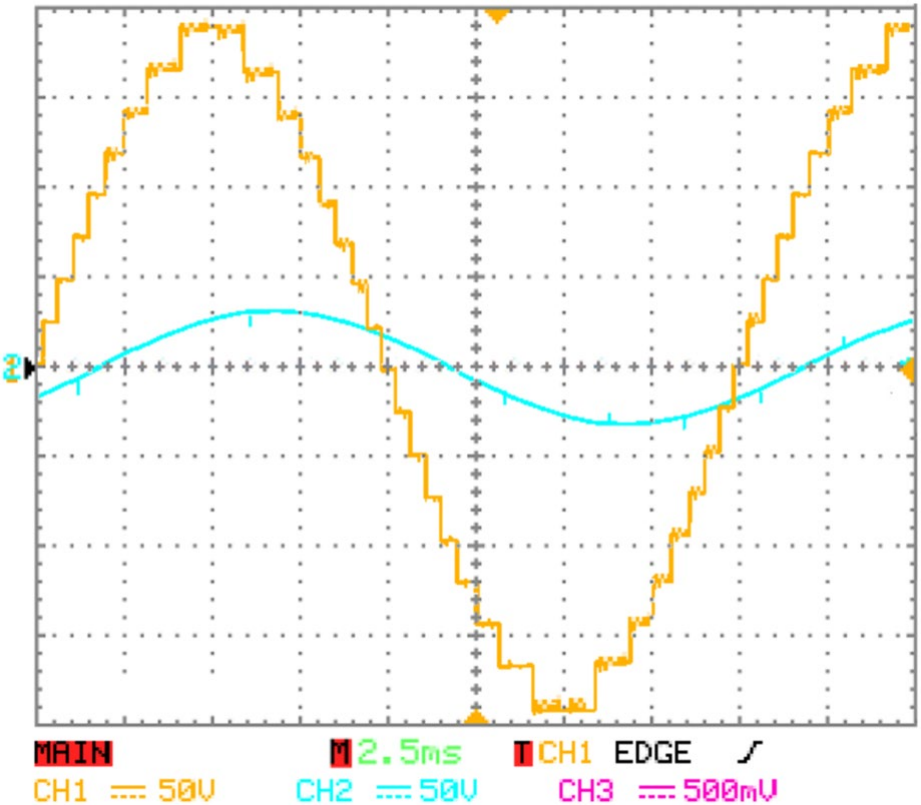
Both the load voltage and current (50V/Div, 50A/Div and 2.5ms/Div).

In Fig. 16, both the load voltage and current are shown. For better visibility, the current trace is magnified 10 times. The inherent ohmic-inductive nature of the load causes the current to lag the voltage in phase.

The measured THD values of 5% for the 9-level configuration and 2% for the 17-level configuration are well within the acceptable limits defined by IEEE Standard 519 for grid-connected applications. These values are consistent with the theoretical expectation that increasing the number of voltage levels reduces harmonic distortion, and they validate the effectiveness of the selective harmonic elimination technique employed in this work.

## Limitations of the proposed topology

While the proposed STUI topology offers significant advantages in terms of reduced switch count and improved output voltage quality, several limitations should be considered for practical implementations:

1. *Requirement for Multiple Isolated DC Sources*: Similar to many cascaded multilevel inverters, the proposed topology requires multiple isolated DC voltage sources to generate the desired output levels. The need for isolated sources also necessitates multi-winding transformers or separate rectifier units, which can impact the overall system footprint and efficiency.
2. *Non-uniform voltage stress distribution*: Although the peak inverse voltage (PIV) of the proposed topology is competitive compared to existing designs, the voltage stress is not uniformly distributed across all semiconductor switches. Some switches, particularly those involved in bidirectional current paths, experience higher voltage stress than others. This non-uniform distribution may require switches with different voltage ratings, potentially complicating component selection and thermal management. However, it should be noted that in cascaded implementations with higher number of levels, this voltage stress becomes more evenly distributed, mitigating this concern.
3. *Increased control complexity for higher levels*: As the number of voltage levels increases for solving equations grows significantly. The number of switching angles that must be calculated increases proportionally with the number of levels, and finding optimal solutions for highly nonlinear equations becomes increasingly challenging. This may require more sophisticated optimization algorithms or look-up tables with large memory requirements. In symmetric configuration, all sources are equal, while asymmetric configuration requires sources with different voltage ratings. This requirement may increase the system complexity and cost in applications where a single DC source is preferred, such as grid-connected systems or renewable energy integration.
4. *Scalability limitations for very high-level implementations*: Although the proposed topology can theoretically be extended to very high number of levels (e.g., 289-level), practical implementation faces challenges related to DC source tolerances, synchronization issues, and increased system complexity. Small imbalances in DC source voltages can accumulate and degrade the output voltage quality, potentially increasing harmonic distortion beyond acceptable limits.

## Conclusions

This paper proposed a novel multilevel inverter structure named STUI (Series Two Unit Inverter), along with its generalized cascaded configuration for both symmetric and asymmetric operation. The proposed topology aims to address the key challenges in multilevel inverter design, particularly component count reduction and cost optimization, while maintaining high output voltage quality. The main contributions and findings of this work can be summarized as follows:

1. *Structural innovation*: The STUI topology introduces a flexible architecture that minimizes the number of active components in the current conduction path at any instant, thereby reducing conduction losses. The structure inherently supports both symmetric operation (using equal DC sources) and asymmetric operation (using different DC source magnitudes), providing designers with flexibility to prioritize either simplicity or component efficiency.
2. *Comprehensive mathematical analysis*: Optimal configurations were derived considering key performance parameters, including the number of switching devices, number of DC voltage sources, achievable output voltage levels, and total standing voltage (PIV).
3. *Experimental validation*: The feasibility and performance of the proposed topology were validated through both simulation and experimental prototypes:
  *Symmetric operation*: A 9-level inverter prototype was implemented and tested, confirming the expected output voltage waveform quality with a THD of 5%.
  *Asymmetric operation*: A 17-level inverter prototype was constructed, achieving a THD of 2% and demonstrating the effectiveness of the selective harmonic elimination technique.
4. *Comparative advantage*: Benchmarking against recent state-of-the-art topologies published in the last five years revealed several advantages of the proposed STUI:
  Lower switch count for the same number of voltage levels
  Competitive PIV values indicating reduced voltage stress on semiconductor devices
  Favorable LSR and LSEMR indices, confirming efficient component utilization
  Reduced number of conducting switches per voltage level, contributing to lower conduction losses
5. *Design flexibility*: The proposed structure offers versatility through multiple voltage source selection algorithms for asymmetric operation, including binary and trinary progressions, enabling optimization for different application requirements.

While the proposed STUI topology demonstrates promising results, several avenues for future research remain open:

1. Reliability and fault-tolerant operation.
2. Advanced modulation techniques.
3. Optimization algorithms for selective harmonic elimination.
4. Integration with renewable energy sources.

## Data Availability

No external datasets were generated or analyzed during the current study. Simulation data and experimental results supporting the findings are available from the corresponding author upon reasonable request.

## References

[1] Baier, C. R. et al. FCS-MPC without steady-state error applied to a grid-connected cascaded H-bridge multilevel inverter. *IEEE Trans. Power Electron.* **36**(10), 11785–11799. 10.1109/TPEL.2021.3076872 (2021).

[2] Das Biswas, S. et al. Enhanced power quality with 15-level reduced switch count multilevel inverter-based shunt active power filter. *Results. Eng.* **27**, 106543. 10.1016/j.rineng.2025.106543 (2025).

[3] Sahli, A., Krim, F., Laib, A. & Talbi, B. Model predictive control for single-phase active power filter using modified packed U-cell (MPUC5) converter. *Electr. Power Syst. Res.* **180**, 106139. 10.1016/j.epsr.2019.106139 (2020).

[4] Banaei, M. R. & Salary, E. Application of multi-stage converter in distributed generation systems. *Energy Convers. Manag.* **62**, 76–83. 10.1016/j.enconman.2012.04.005 (2012).

[5] Liu, J., Wu, J., Zeng, J. & Guo, H. A novel nine-level inverter employing one voltage source and reduced components as high-frequency AC power source. *IEEE Trans. Power Electron.* **32**(4), 2939–2947. 10.1109/TPEL.2016.2587744 (2017).

[6] Gupta, K. K., Ranjan, A., Bhatnagar, P., Sahu, L. K. & Jain, S. Multilevel inverter topologies with reduced device count: A review. *IEEE Trans. Power Electron.* **31**(1), 135–151. 10.1109/TPEL.2015.2405012 (2016).

[7] Vijeh, M., Rezanejad, M., Samadaei, E. & Bertilsson, K. A general review of multilevel inverters based on main submodules: Structural point of view. *IEEE Trans. Power Electron.* **34**(10), 9479–9502. 10.1109/TPEL.2019.2890737 (2019).

[8] Babaei, E. & Hosseini, S. H. New cascaded multilevel inverter topology with minimum number of switches. *Energy Convers. Manag.* **50**(11), 2761–2767. 10.1016/j.enconman.2009.06.032 (2009).

[9] Hinago, Y. & Koizumi, H. A single-phase multilevel inverter using switched series/parallel DC voltage sources. *IEEE Trans. Ind. Electron.* **57**(8), 2643–2650. 10.1109/TIE.2009.2038954 (2010).

[10] Babaei, E., Kangarlu, M. F., Sabahi, M. & Pahlavani, M. R. A. Cascaded multilevel inverter using sub-multilevel cells. *Electr. Power Syst. Res.* **96**, 101–110. 10.1016/j.epsr.2012.10.004 (2013).

[11] Ajami, A., Oskuee, M. R. J., Mokhberdoran, A. & Khosroshahi, M. T. Advanced cascade multilevel converter with reduction in number of components. *J. Electr. Eng. Technol.* **9**(1), 127–135. 10.5370/JEET.2014.9.1.127 (2014).

[12] Oskuee, M. R. J., Salary, E. & Najafi-Ravadanegh, S. Creative design of symmetric multilevel converter to enhance the circuit’s performance. *IET Power Electron.* **8**(1), 96–102. 10.1049/iet-pel.2014.0053 (2015).

[13] Bektas, Y. Real-time control of selective harmonic elimination in a reduced switch multilevel inverter with unequal DC sources. *Ain Shams Eng. J.* **15**, 102719. 10.1016/j.asej.2024.102719 (2024).

[14] Raghava Sharma, P. V. V. Novel cascaded tilt fractional-order integral derivative with a proportional integral for harmonics mitigation in 31-level multilevel inverter. *Comput. Electr. Eng.* **123**, 110280. 10.1016/j.compeleceng.2025.110280 (2025).

[15] Ebrahimi, J., Babaei, E. & Gharehpetian, G. B. A new topology of cascaded multilevel converters with reduced number of components for high-voltage application. *IEEE Trans. Power Electron.* **26**, 3109–3118. 10.1109/TPEL.2011.2127486 (2011).

[16] Babaei, E., Sheermohammadzadeh, S. & Sabahi, M. Improvement of multilevel inverter topology using series and parallel connections of DC voltage sources. *Arab. J. Sci. Eng.* **39**(2), 1117–1127. 10.1007/s13369-013-0647-5 (2014).

[17] Odeh, C. I., Obe, E. S. & Ojo, O. Topology for cascaded multilevel inverter. *IET Power Electron.* **9**(5), 921–929. 10.1049/iet-pel.2015.0131 (2016).

[18] Samadaei, E., Gholamian, S. A., Sheikholeslami, A. & Adabi, J. An envelope type (E-type) module: Asymmetric multilevel inverters with reduced components. *IEEE Trans. Ind. Electron.* **63**(11), 7148–7156. 10.1109/TIE.2016.2529563 (2016).

[19] Samadaei, E., Sheikholeslami, A., Gholamian, S. A. & Adabi, J. A square T-type (ST-type) module for asymmetrical multilevel inverters. *IEEE Trans. Power Electron.* **33**(2), 987–996. 10.1109/TPEL.2017.2675381 (2018).

[20] Majumdar, S., Mahato, B. & Jana, K. C. Implementation of an optimum reduced components multicell multilevel inverter (MC-MLI) for lower standing voltage. *IEEE Trans. Ind. Electron.* **67**(4), 2765–2775. 10.1109/TIE.2019.2912780 (2020).

[21] Dhanamjayulu, C., Padmanaban, S., Holmnielsen, J. B. & Blaabjerg, F. Design and implementation of a single-phase 15-level inverter with reduced components for solar PV applications. *IEEE Access* **9**, 581–594. 10.1109/ACCESS.2020.3047324 (2021).

[22] Kakar, S. et al. New asymmetrical modular multilevel inverter topology with reduced number of switches. *IEEE Access* **9**, 27627–27637. 10.1109/ACCESS.2021.3058534 (2021).

[23] Peddapati, S. A new fault-tolerant multilevel inverter structure with reduced device count and low total standing voltage. *IEEE Trans. Power Electron.* **37**(7), 8333–8344. 10.1109/TPEL.2022.3152109 (2022).

[24] Mondol, M. H., Biswas, S. P., Rahman, M. A. & Islam, M. R. A new hybrid multilevel inverter topology with level-shifted multicarrier PWM technique for harvesting renewable energy. *IEEE Trans. Ind. Electron.* **69**(2), 2574–2585. 10.1109/TIE.2022.3157364 (2022).

[25] Hosseinzadeh, M. A., Sarebanzadeh, M., Babaei, E. & Rivera, M. New generalized circuits for single-phase multisource multilevel power inverter topologies. *IEEE Trans. Power Electron.* **38**(6), 6823–6830. 10.1109/TPEL.2023.3238656 (2023).

[26] Sirohi, V., Saggu, T. S., Kumar, J. & Gill, B. Implementation of a novel multilevel inverter topology with minimal components—an experimental study. *IEEE Can. J. Electr. Comput. Eng.* **47**(1), 7–18. 10.1109/ICJECE.2024.3357679 (2024).

[27] Anand, R. & Mandal, R. K. An efficient and high gain switched-capacitor based multi-level inverter. *Eng. Res. Express.* **4**(3), 035019. 10.1088/2631-8695/ac84c6 (2022).

[28] Meraj, S. T. et al. A pencil shaped 9-Level multilevel inverter with voltage boosting ability: Configuration and experimental investigation. *IEEE Access* **10**, 9844712 (2022).

[29] Alaas, Z. A new active neutral point clamped (ANPC) nine-level inverter topology with low energy storage switched capacitors. *Sci. Rep.* **15**, 7031. 10.1038/s41598-025-87302-2 (2025). 40016331 10.1038/s41598-025-87302-2 PMC11868572

[30] Saif, B., Sarwar, A., Ahmad, S., Lu, S.-D. & Liu, H.-D. A single-phase seven-level switched capacitor with common ground inverter and improved phase-shift modulation technique. *Sci. Rep.* **15**, 4209. 10.1038/s41598-025-86180-y (2025). 39905085 10.1038/s41598-025-86180-y PMC11794881

[31] Anand, R. & Mandal, R. K. A novel 13-level switched-capacitor step-up inverter with reduced component count. *Int. J. Circ. Theory Appl.* **52**(10), 5295–5317. 10.1002/cta.4002 (2024).

[32] Anand, R. & Mandal, R. K. A five-level (5-L) double gain inverter for grid-connected and photovoltaic applications. *Electr. Eng.* **106**(5), 5447–5460. 10.1007/s00202-024-02282-2 (2024).

[33] Anand, R., Mandal, R. K. & Choudhary, A. A novel seven-level inverter with high gain and reducing spike current capabilities. *Int. J. Circuit Theory Appl.* **53**(5), 2362–2380 (2025).

[34] Hasan, M. M., Mekhilef, S. & Ahmed, M. Three-phase hybrid multilevel inverter with less power electronic components using space vector modulation. *IET Power Electron.* **7**(5), 1256–1265. 10.1049/iet-pel.2013.0396 (2014).

[35] AlZoubi, S. & Ghazi Batarseh, M. Optimized phase disposition sinusoidal pulse width modulation classical multilevel inverters using curve fitting techniques and genetic algorithm. *Comput. Electr. Eng.* **125**, 110464. 10.1016/j.compeleceng.2025.110464 (2025).

